# Cow Behavior Recognition Method Based on Multi-Source Perceptual Information Fusion

**DOI:** 10.3390/vetsci13090856

**Published:** 2026-08-24

**Authors:** Xiuyan Zhao, Hongzheng Sun, Kaixing Zhang, Junchi Sun, Yilong Lin, Jianzhu Liu

**Affiliations:** 1 College of Information Science and Engineering, Shandong Agricultural University, Tai’an 271018, China; 2 College of Mechanical and Electronic Engineering, Shandong Agricultural University, Tai’an 271018, China; 3 College of Computer Science and Engineering, Jining University, Qufu 273155, China; 4 College of Veterinary Medicine, Shandong Agricultural University, Tai’an 271018, China

**Keywords:** dairy cow, inertial measurement unit, ultra-wideband ranging, wearable sensors, deep learning, behavior classification

## Abstract

This study proposes a multi-source perceptual information fusion method to improve the accuracy and stability of dairy cow behavior monitoring. Existing machine vision approaches are often affected by lighting conditions, occlusion, and complex cowshed environments, while single wearable inertial measurement unit (IMU) devices may confuse similar behaviors such as eating, ruminating, standing, and lying. To address these limitations, a wireless collar was developed to synchronously collect nine-axis IMU data and ultra-wideband (UWB) ranging data in real time. Combined with manual behavioral observations, a dataset covering seven behaviors—eating, ruminating, standing, lying, drinking, sleeping, and lateral trunk contact—was constructed. By integrating neck-motion features extracted from the IMU data with spatial-distance features obtained from the UWB data, an IMU–UWB dual-branch fusion model was developed to automatically classify dairy cow behaviors. The results indicate that the proposed method can effectively reduce confusion among similar behaviors and improve the recognition of behaviors with limited samples. This approach enables more comprehensive assessment of dairy cows’ daily activities and health status, providing technical support for health monitoring, early disease warning, and intelligent dairy farm management.

## Simple Summary

Monitoring dairy cow behavior can help farmers promptly identify changes related to animal health and welfare. However, manual observation is time-consuming, while systems relying on a single type of sensor may have difficulty distinguishing between behaviors with similar movement patterns. In this study, a wearable collar integrating motion sensing and wireless distance measurement was developed to improve the accuracy of automatic dairy cow behavior recognition. Data were collected on a commercial dairy farm and covered seven behaviors: eating, ruminating, standing, lying, drinking, sleeping, and lateral trunk contact. A computational model was further developed to integrate motion-related and location-related information. In experimental evaluations, the proposed model achieved an overall recognition accuracy of 98.61% and effectively reduced confusion among behaviors with similar movement patterns. This approach provides a practical method for continuous monitoring of dairy cow activities and may support the early detection of abnormal behavior, improved animal welfare assessment, and more efficient dairy farm management.

## 1. Introduction

Dairy cow behavior is an important indicator of animal welfare and health [1]. In the context of climate change, livestock reproductive performance and productivity are increasingly being affected [2]. Automatic monitoring and classification of cow behaviors can facilitate the early detection of health or welfare problems. For example, reduced eating and rumination time may be associated with digestive disorders [3,4], whereas prolonged lying time may indicate disease or discomfort [5]. Accurate recognition of dairy cow behaviors is a prerequisite for reliably quantifying their duration, frequency, and temporal patterns, which can subsequently be used to detect deviations from individual normal activity patterns. Traditional behavior monitoring mainly relies on manual observation, which is labor-intensive, subjective, and lacks real-time capability [6]. Therefore, continuous and accurate automatic recognition of dairy cow behavior is essential for improving management efficiency and reducing labor costs in large-scale farming [7,8].

Dairy cow behavior detection methods can be broadly classified into non-contact and contact-based approaches [9]. Non-contact methods, such as cameras and video surveillance systems [10,11,12], acquire image or video data without direct contact with animals and use machine vision techniques for behavior recognition [13]. Although these methods enable continuous monitoring of group behaviors, their performance is easily affected by illumination changes, occlusion, camera angle, and complex barn environments [14,15,16]. They also require considerable storage and computational resources, which limits their practical application in real farming scenarios.

For contact-based behavior detection, wearable sensors have become an important component in the development of precision livestock farming because of their relatively low cost [17,18]. In particular, inertial measurement units (IMUs) are widely used because of their small size, low power consumption, and ease of deployment [19,20]. By collecting multi-axis acceleration and angular velocity data from the neck or trunk of dairy cows and combining them with machine learning or deep learning methods, multiple behaviors can be automatically recognized [21,22]. However, IMU-only methods may suffer from recognition confusion when different behaviors exhibit similar motion patterns, thereby reducing accuracy [23]. To address this issue, multi-source information fusion has been explored [24]. For example, Zhang Y. et al. combined IMU and image data for classifying behaviors such as drinking, standing, and walking, achieving improved accuracy [25]. Wang Liwei et al. fused sound and IMU data to improve rumination recognition [26]. Yuan Wenqiang et al. integrated triaxial acceleration, temperature, and heart rate data for behavior classification and health assessment [27]. Liu Xinpeng et al. combined IMU and ultra-wideband (UWB) data for behavior classification and movement analysis [28]. In fact, UWB positioning can provide high-precision location information [29], reflecting the activity range and spatial state of dairy cows and offering complementary information for behavior recognition.

Overall, IMU-only wearable recognition methods are prone to confusion among similar behaviors [30,31,32], while existing multimodal approaches often suffer from complex models, difficult deployment, or insufficient use of auxiliary information. To overcome these limitations, this study proposes a behavior recognition method based on multi-source sensing information fusion. The proposed method performs joint modeling of multi-source sensing data through an end-to-end fusion framework, aiming to achieve accurate and robust recognition of dairy cow behaviors.

## 2. Materials and Methods

### 2.1. Experimental Materials

The experimental device used in this study was a dairy cow collar system consisting of a microprocessor (Espressif Systems, Shanghai, China), a nine-axis IMU (Yahboom Technology Co., Ltd., Shenzhen, China), a UWB module (Guangzhou Networking Technology Co., Ltd., Guangzhou, China), and a power supply module. The IMU was used to measure neck-motion- and posture-related signals, including triaxial acceleration, angular velocity, and orientation angles, while the UWB module was used to measure the distances between the collar-mounted node and the fixed tag nodes. All sensor data were transmitted to the host computer via Bluetooth.

**Figure 1:**
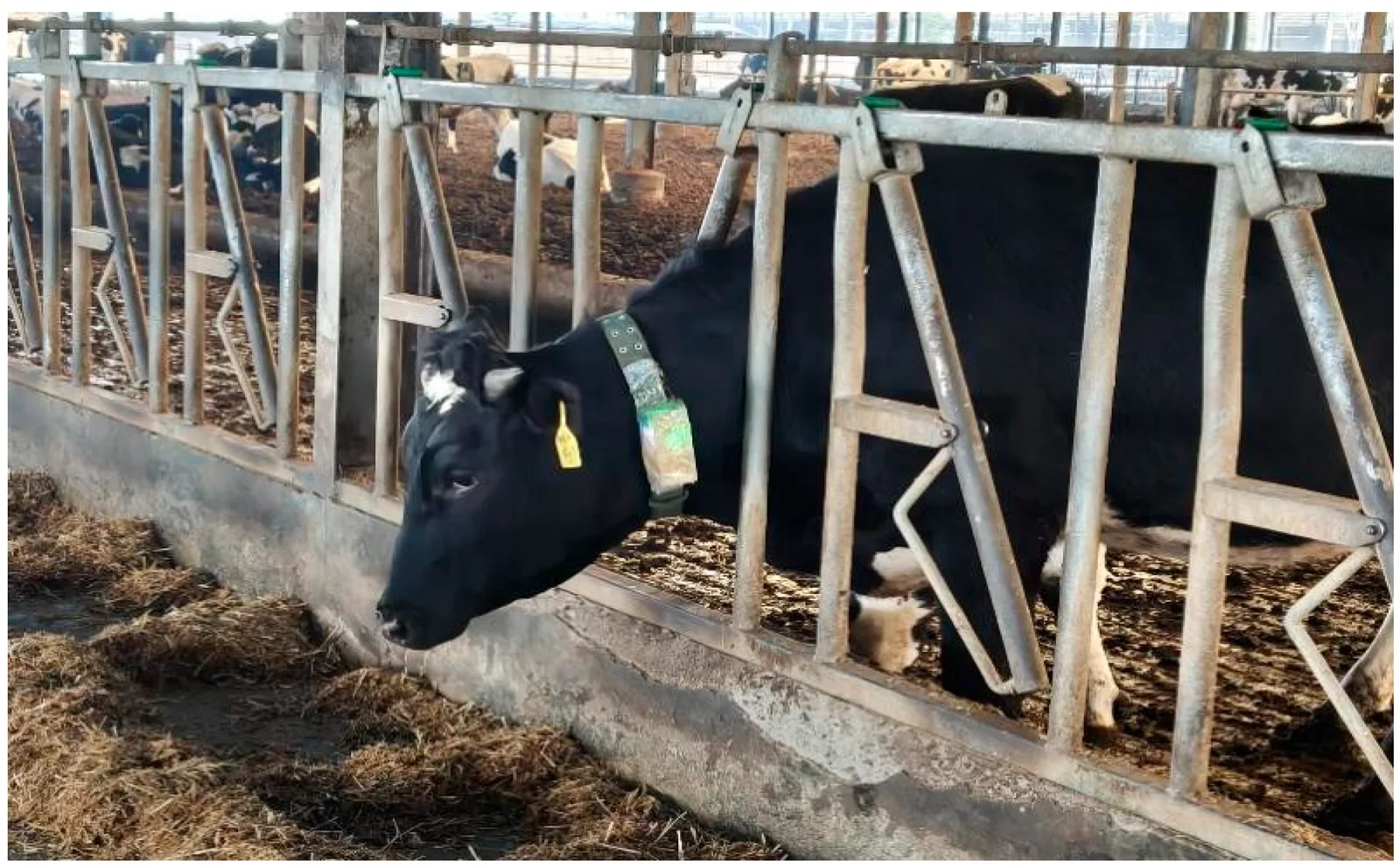
Experimental apparatus photo.

The microprocessor was an ESP32-WROOM-32E wireless microcontroller manufactured by Espressif Systems, featuring high processing performance, convenient wireless communication, and low power consumption. The IMU was a Yahboom nine-axis IMU module, which communicated with the ESP32 via an inter-integrated circuit (I2C) bus. The UWB module was a D-DWM-PG1.7, which communicated with the ESP32 via a universal asynchronous receiver-transmitter (UART). The power supply module was a portable power bank. The microprocessor, nine-axis IMU sensor, UWB master base-station sensor, and power supply module were enclosed in a customized acrylic case and fixed to the neck of the dairy cow, as shown in Figure 1.

### 2.2. Experimental Data Acquisition

**Figure 2:**
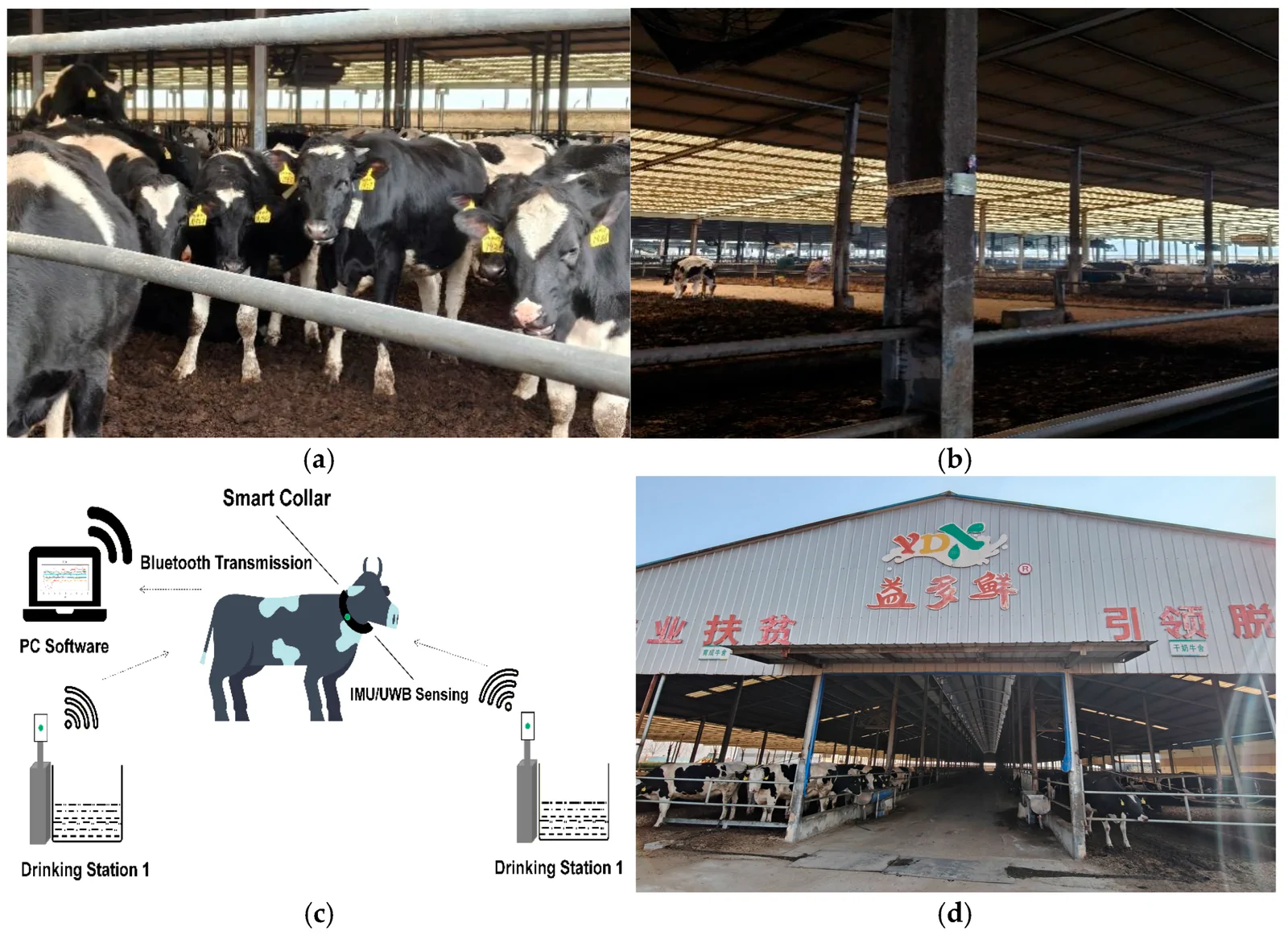
Data acquisition diagram. (**a**) Experimental site images; (**b**) Schematic diagram of tag installation; (**c**) Data acquisition diagram; (**d**) Overall view of the barn.

In March 2026, data collection experiments were conducted at Shandong Wanxiang Animal Husbandry Co., Ltd. in Jining, China, a large-scale commercial dairy farm with more than 1000 dairy cows. The cows were housed in a loose-housing barn. Four healthy Holstein dairy cows were selected for the experiment. Each cow wore a collar around its neck, and two ultra-wideband (UWB) tag nodes were installed near the water troughs on both sides of the experimental area. The overall view of the barn, the experimental site, the UWB tag installation, and the data acquisition system are presented in Figure 2.

During cow activity within the breeding area, the nine-axis IMU sensor collected motion data from the node in real time, including triaxial acceleration (g), angular velocity (°/s), and angle (°). Meanwhile, the UWB master base station obtained the distances between the current node and the two tag nodes using a ranging algorithm, with distances measured in cm. The ESP32 read the motion data from the IMU sensor and the ranging data from the UWB module, packaged and processed the collected data uniformly, and wirelessly transmitted them to the host computer via the Bluetooth communication module.

Considering the power consumption of Bluetooth communication, the IMU sampling frequency was set to 20 Hz after experimental validation, whereas the UWB sampling frequency was fixed at 100 Hz. During data acquisition, each IMU record was associated with the latest UWB data and output together to achieve data alignment. To evaluate the generalization performance of the collar, data were collected from four healthy dairy cows freely moving in the same breeding area for six consecutive days, resulting in a total of 1.2 × 10^7^ data records. During each daily data collection session, two trained researchers continuously observed the behaviors of the four dairy cows and recorded them manually in real time using stopwatches. The corresponding sensor data were then annotated according to these observations to generate ground-truth labels for model training and evaluation. To reduce annotation uncertainty, periods involving behavioral transitions, temporary visual obstruction, or behaviors that could not be clearly assigned to one of the seven predefined categories were treated as ambiguous segments and excluded from the valid dataset.

### 2.3. Sample Set Construction

Based on the collected 1.2 × 10^7^ raw data records, 402,729 valid samples corresponding to seven target behaviors were extracted as the experimental dataset, followed by denoising preprocessing. The raw data were stored in comma—separated values (CSV) format and consisted of 12 columns: 11 feature variables and one class label in the final column. The 11 features and corresponding labels at all time points were extracted to form point-level time-series data. Z-score normalization was applied to eliminate differences in feature scales, and the calculation formula is as follows:

(1) $$z=\frac{x-\mu }{\sigma }$$

where x is the original data value, μ is the population mean, and σ is the population standard deviation.

After preprocessing, the time-series signals were segmented using a sliding-window method, with a window length of 40 sampling points and a step size of 20 sampling points. Window labels were assigned using a majority voting strategy, in which the class with the highest frequency within each window was selected as the window label. In addition, a purity threshold of 0.8 was applied, and only window samples in which the dominant class accounted for at least 80% were retained.

Finally, 20,082 samples were obtained, and the sample distribution by class is presented in Table 1.

Since IMU and UWB data exhibit clear differences in information representation, IMU data mainly describe local motion characteristics, whereas UWB data provide complementary spatial distance information. The two types of information are highly complementary. Therefore, relying solely on IMU data is insufficient to comprehensively characterize dairy cow behaviors. Based on this, an IMU–UWB dual-branch fusion model was further constructed.

### 2.4. Dairy Cow Behavior Classification Method

#### 2.4.1. IMU–UWB Dual-Branch Fusion Model

**Figure 3:**
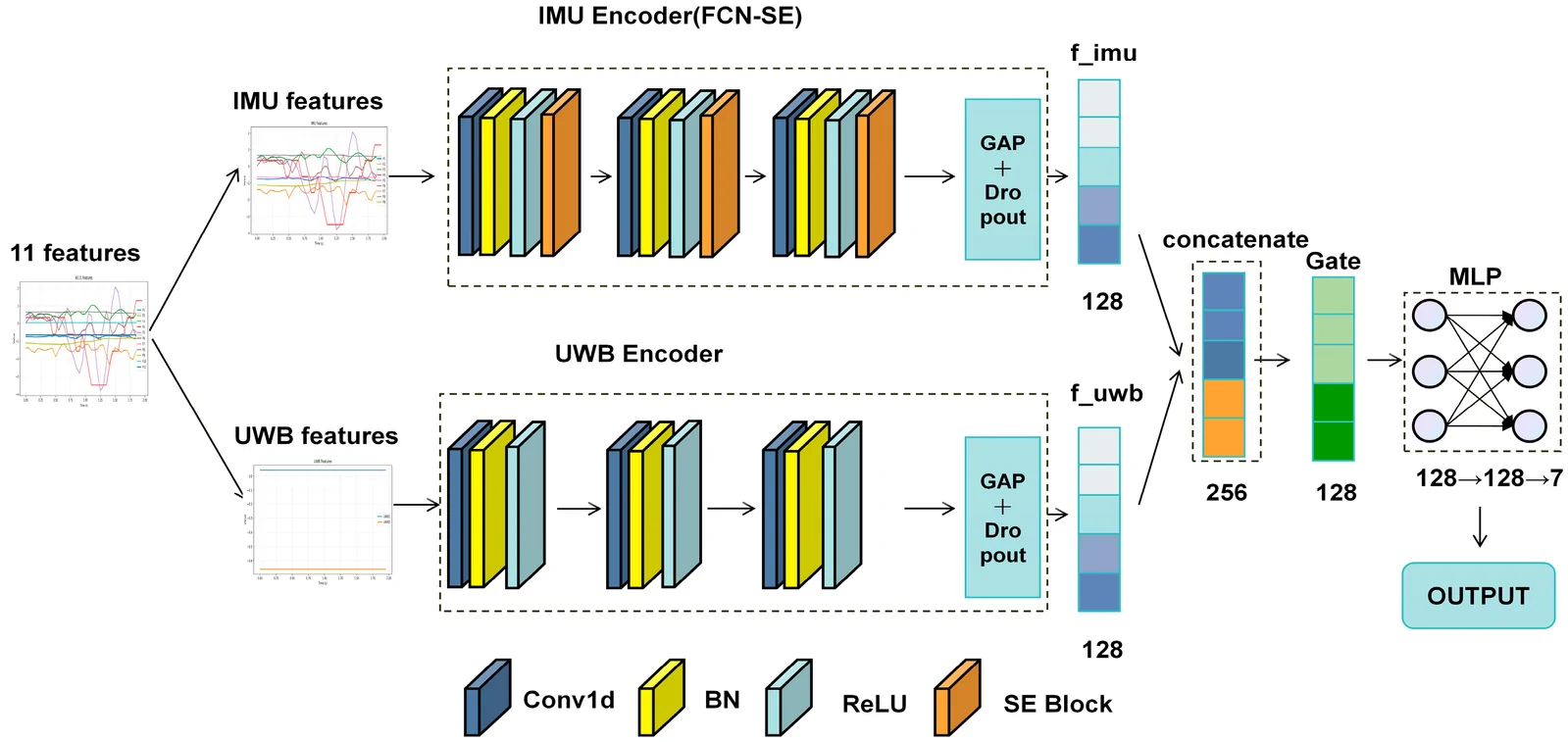
Dual- branch fusion model.

To fully exploit the complementary information contained in multi-source sensor signals for dairy cow behavior recognition, this study proposes a dual-branch fusion model that improves recognition accuracy while maintaining a lightweight architecture. Specifically, the model takes temporal features as input and divides them into an IMU branch and a UWB branch. The IMU branch employs a fully convolutional network with squeeze-and-excitation modules (FCN-SE) as its encoder to extract the primary temporal dynamic features, and pretrained weights are used to initialize the parameters of this branch, thereby enhancing its feature representation capability. The UWB branch adopts a lightweight one-dimensional convolutional encoder to extract auxiliary features, thereby complementing the spatial feature information. Subsequently, the features from the two branches are concatenated and adaptively fused through a gating mechanism. During training, a staged optimization strategy is adopted. First, the pretrained IMU branch is frozen, and only the UWB branch, fusion module, and classifier are optimized. Then, the IMU branch is unfrozen for joint fine-tuning, thereby improving the overall recognition performance while maintaining the stability of the main branch. Meanwhile, to mitigate the influence of the imbalanced sample distribution among behavioral categories, a class-weighted cross-entropy loss was adopted during model training, assigning greater weights to minority-sample categories. The architecture of the dual-branch fusion model is shown in Figure 3.

#### 2.4.2. IMU Branch Pretraining

**Figure 4:**
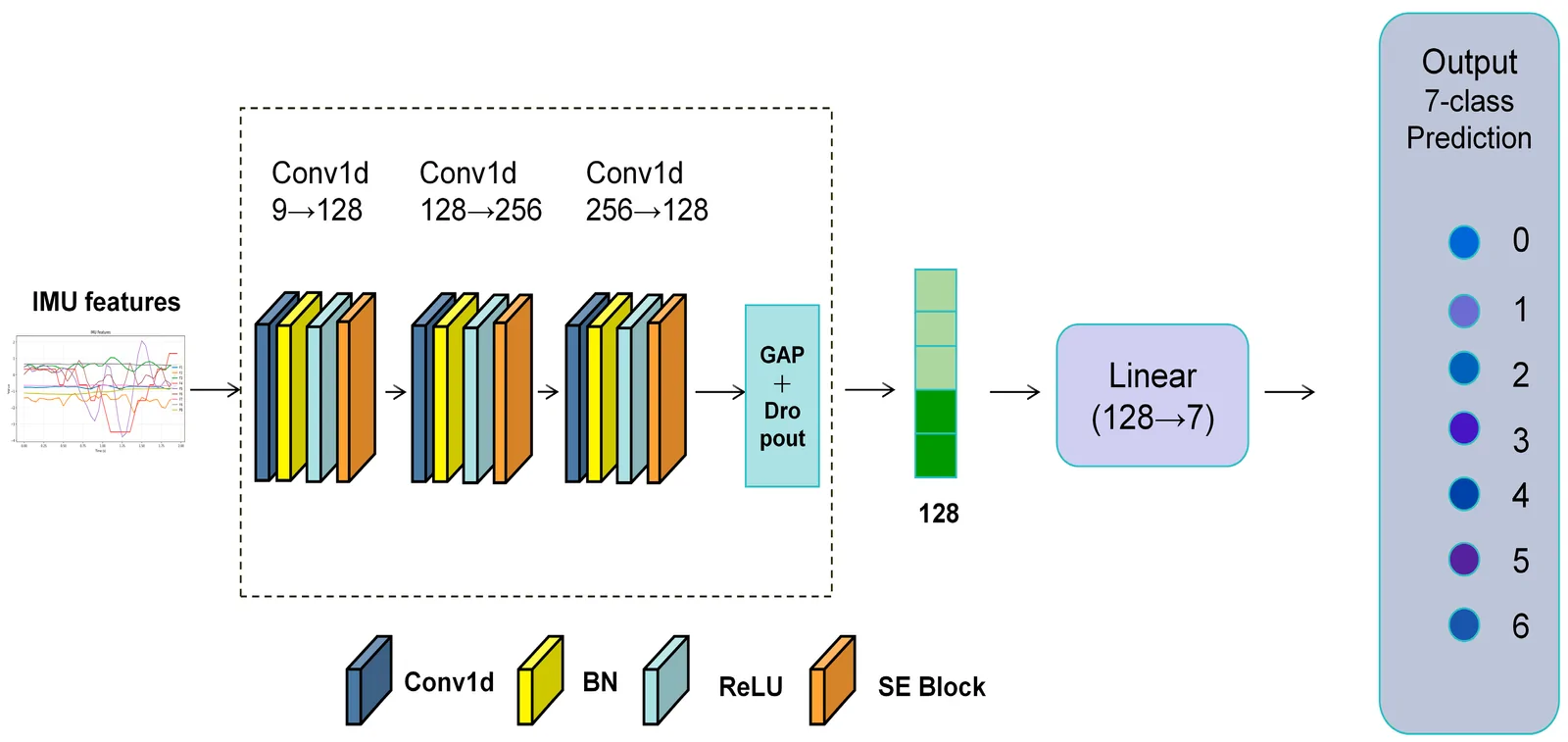
Network structure of FCN-SE.

In multi-source dairy cow behavior recognition tasks, model performance largely depends on the effectiveness of feature extraction in each branch. IMU signals serve as a key data source in this study. If all branches are directly trained jointly, the randomly initialized network parameters may lead to unstable feature representations during the early stage of training, thereby affecting the overall model performance. Therefore, this study first constructs an FCN-SE pretraining model based on IMU data to provide a stable feature foundation for the subsequent dual-branch fusion model. Specifically, the model first rearranges the dimensions of the input IMU time-series data. Subsequently, the data are sequentially passed through three feature extraction modules, each consisting of a one-dimensional convolutional block and an SE (Squeeze-and-Excitation) attention module. The convolutional block, denoted as CBR, is composed of a convolutional layer (Conv), a batch normalization layer (BN), and a rectified linear unit (ReLU) activation function, and is used to extract local temporal features layer by layer. After each convolutional layer, an SE attention module is introduced to recalibrate the convolutional outputs along the channel dimension, enhancing the response of important channel features and thereby improving the model’s ability to represent key behavioral patterns. The structure of the FCN-SE model is shown in Figure 4.

#### 2.4.3. SE Attention Mechanism

To enhance the IMU branch’s ability to represent multichannel temporal features, this study introduces the SE channel attention mechanism after the FCN-based convolutional feature extraction module. IMU signals consist of multiple types of sensor information, including triaxial acceleration, angular velocity, and angle data. Different channels contribute differently to the representation of dairy cow behavioral states.

**Figure 5:**
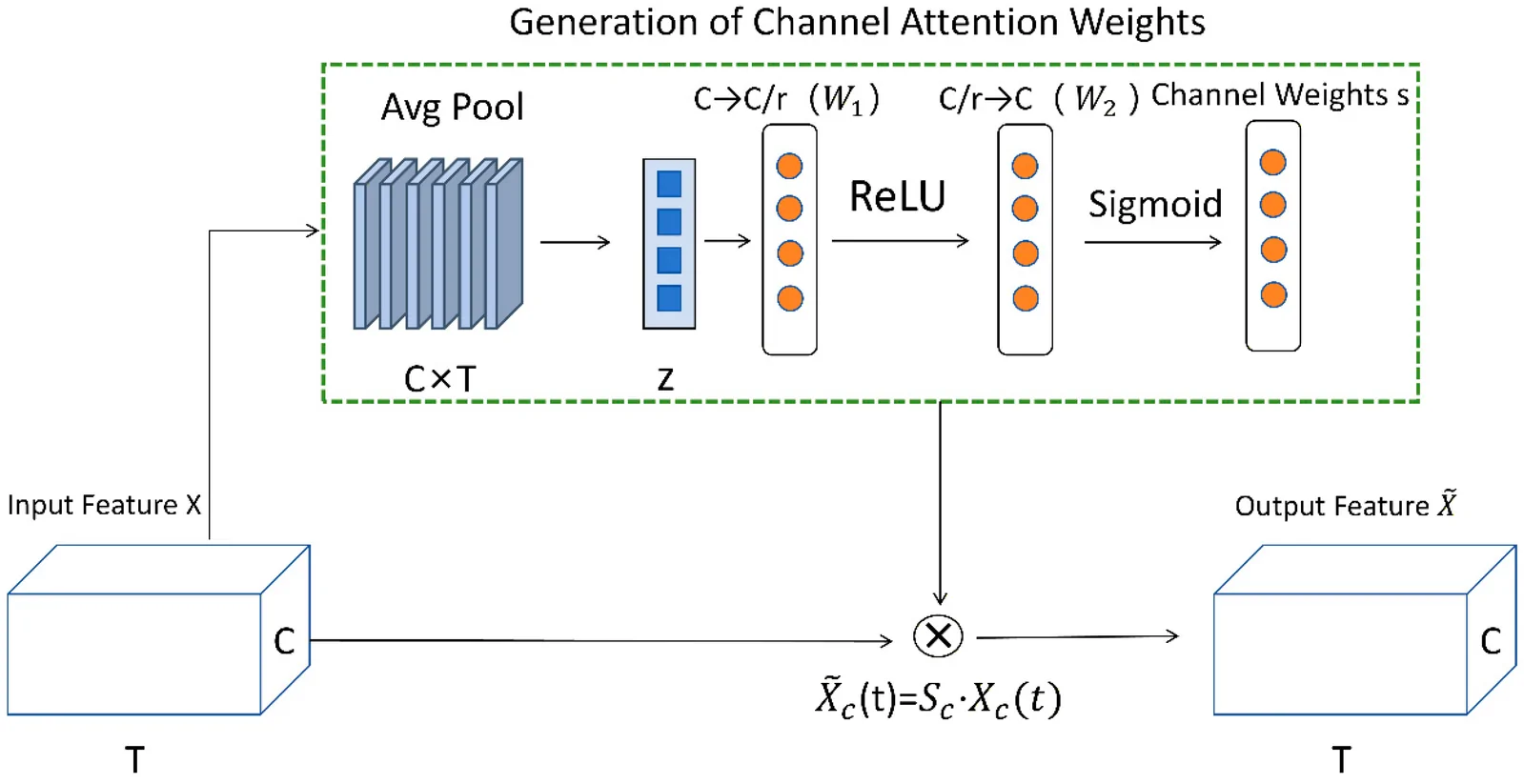
Network structure of SE.

Traditional convolutional networks often assign equal importance to features from different channels during feature extraction, making it difficult to highlight key channel information. The SE attention mechanism explicitly models inter-channel dependencies and adaptively learns the importance weights of different feature channels, thereby enhancing the representation of key behavioral features while suppressing interference from redundant information. The schematic diagram of the SE network structure is shown in Figure 5.

Let the input feature be denoted as X. First, global average pooling is applied along the temporal dimension to compress each channel, obtaining the channel descriptor vector z. Then, z is fed into a bottleneck structure composed of two fully connected layers, and the channel weight vector s is generated through the ReLU and Sigmoid activation functions. Finally, the channel weights are multiplied with the original input features channel by channel to obtain the recalibrated output feature $\tilde{X}$. The calculation process of the SE attention mechanism is as follows:

(2) $$z_{c}=\frac{1}{T}\sum_{t=1}^{T}x_{c}\left(t\right)$$

(3) $$s=\sigma (W_{2}\delta (W_{1}z))$$

(4) $$\tilde{x_{c}}=s_{c}\cdot x_{c}(t)$$

where $x_{c}\left(t\right)$ denotes the feature value of channel c at time t; $z_{c}$ denotes the global descriptor of channel c; $W_{1}$ and $W_{2}$ denote the weights of the fully connected layers; $s_{c}$ denotes the channel weight; $\tilde{x_{c}}$ denotes the recalibrated feature; δ(·) and σ(·) denote the ReLU and Sigmoid functions, respectively.

#### 2.4.4. Gating Mechanism-Based Dual-Branch Feature Fusion

**Figure 6:**
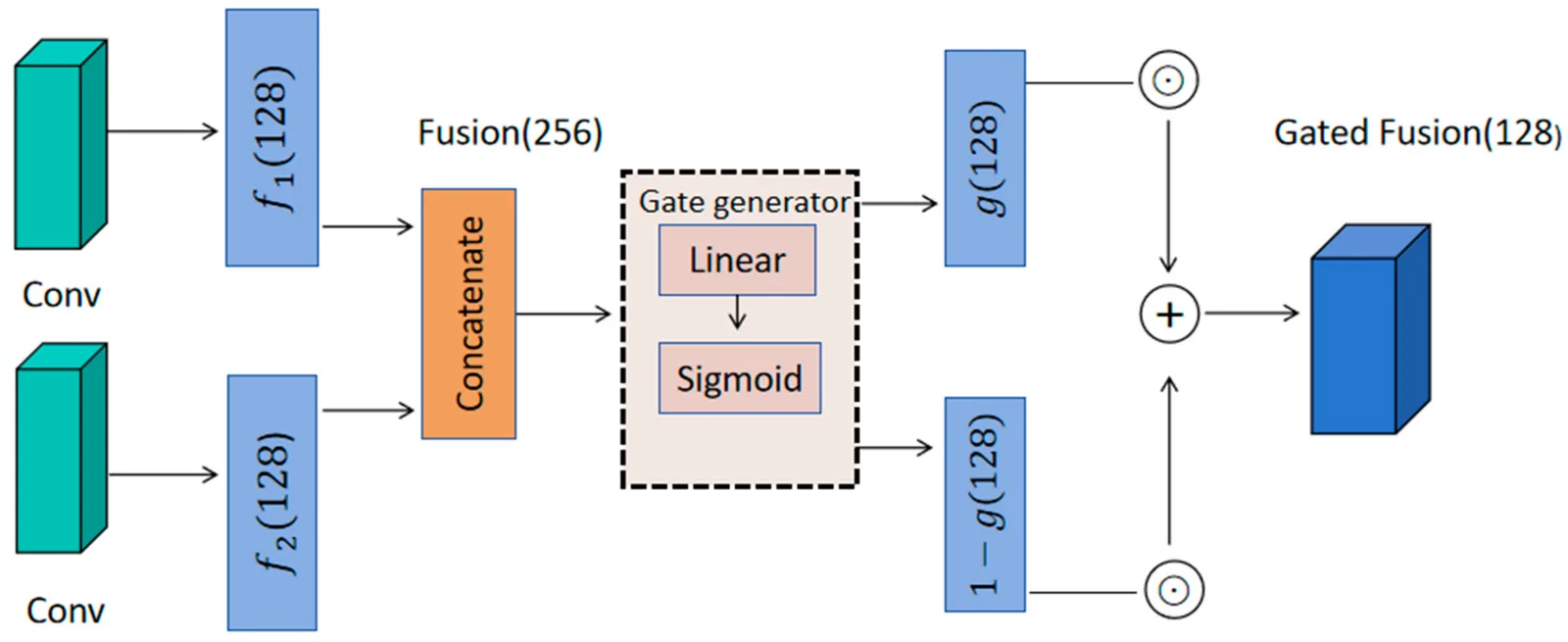
Gated fusion mechanism.

IMU signals mainly reflect temporal motion variations during dairy cow behaviors, while UWB ranging information provides spatial distance features. These two types of information are complementary to a certain extent. To fully utilize different features and avoid redundant interference caused by simple concatenation, this study introduces a gating fusion mechanism into the dual-branch network to adaptively weight and fuse IMU features and UWB features. The gating fusion mechanism is shown in Figure 6.

Let the feature extracted by the IMU branch be denoted as $f_{1}$, and the feature extracted by the UWB branch be denoted as $f_{2}$. The two features are concatenated along the feature dimension to obtain the joint feature ([$f_{1}$; $f_{2}$]). Then, the gating weight g is generated through a linear layer and a Sigmoid function. The calculation formula is as follows:

(5) $$g=\sigma (W_{g}[f_{1};f_{2}]+b_{g})$$

where $W_{g}$ denotes the weight matrix of the gating generation module, $b_{g}$ denotes the bias term of the gating generation module, and σ denotes the sigmoid activation function.

Subsequently, g and 1 − g are used to perform element-wise weighting on the IMU features and UWB features, respectively, and the weighted features are added to obtain the fused feature $f_{c}$. The calculation formula is as follows:

(6) $$f_{c}=g⨀f_{1}+(1-g)⨀f_{2}$$

where $f_{c}$ denotes the fused feature, and ⨀ denotes element-wise multiplication.

Through this mechanism, the model can dynamically adjust the weights of IMU and UWB features according to the input samples, introducing effective spatial information while preserving the primary temporal dynamic features, thereby enhancing the model’s ability to distinguish between samples.

### 2.5. Test Platform and Model Test Index

#### 2.5.1. Testing Platform and Validation Methodology

The experimental platform was established under the Windows 10 operating system. The hardware configuration included an Intel Core i5-10300H processor, 8 GB of memory, and an NVIDIA GeForce GTX 1650 Ti graphics card. The software environment was based on the PyCharm (v2024) integrated development environment, using the Python 3.8 programming language.

In terms of experimental settings, stratified 10-fold cross-validation was used to evaluate the model performance. The dataset was divided into 10 subsets. In each experiment, one subset was selected as the validation set, while the remaining subsets were used to train the model. The model was trained for 50 epochs with a batch size of 64 using the Adam optimizer and the OneCycle learning-rate scheduling strategy. The dropout rate was set to 0.08, and a fixed random seed of 42 was used. This process was repeated 10 times, and the average values of each evaluation metric were taken as the final results to improve the reliability and stability of the evaluation.

#### 2.5.2. Test Evaluation Indicators

To comprehensively evaluate the performance of the proposed IMU-UWB dual-branch fusion model in the dairy cow behavior recognition task, this study constructs a comprehensive evaluation system from two dimensions: classification performance and model complexity.

In terms of classification performance, Precision, Recall, and F1-score are used as the core evaluation metrics to assess the model’s ability to recognize different behavioral categories and its overall classification performance. In terms of model complexity, the number of model parameters, floating-point operations (FLOPs), and model storage size are selected as evaluation metrics. The formulas for Precision, Recall, and F1-score are as follows:

(7) $$Precision=\frac{TP}{TP+FP}$$

(8) $$Recall=\frac{TP}{TP+FN}$$

(9) $$F1=2\times \frac{Precision\times Recall}{Precision+Recall}$$

where TP denotes the number of true positive samples, FP denotes the number of false positive samples, FN denotes the number of false negative samples, and F1 denotes the harmonic mean of Precision and Recall.

## 3. Results

### 3.1. Training Results

To evaluate the overall performance of the proposed dual-branch fusion model, this paper conducts a statistical analysis of the results obtained from 10-fold training. The model performance parameters are shown in Table 2 below.

In terms of classification performance, the model achieved high recognition accuracy in the behavior recognition task. The average accuracy, macro-average recall, and macro-average F1-score reached 98.61%, 99.12%, and 98.93%, respectively. From the perspective of the overall evaluation metrics, the model maintained high accuracy while also achieving a high recall rate, indicating that it can effectively reduce missed detections. Meanwhile, the high F1-score demonstrates that the model has stable overall classification performance.

In terms of model complexity, the total number of parameters was 0.384 M, and the floating-point operations amounted to 0.013 GFLOPs. The small number of model parameters is conducive to reducing training difficulty and lowering the risk of overfitting. The low computational cost and resource usage indicate that the model has high computational efficiency while requiring relatively low memory consumption.

Although the average accuracy, macro-average recall, and macro-average F1-score can characterize the overall performance of the model, they are insufficient to fully reveal its recognition capability across different cow behavior categories. Therefore, this study calculated the precision, recall, and F1-score for seven behavior categories during 10-fold training and averaged the results to evaluate the model’s recognition performance for different behavior categories. The specific results are shown in Table 3.

As shown in Table 3, the model achieved satisfactory recognition performance for all categories of cow behavior. The recognition performance for behaviors such as eating, drinking, and sleeping was particularly outstanding, with F1-scores all exceeding 99%. This indicates that the model can effectively extract the salient features of these behaviors and achieve high-precision recognition. Meanwhile, the model also demonstrated strong recognition capability for behaviors such as rumination, standing, and lying.

In addition, the accuracy, recall, and F1-score for lateral trunk contact all remained at 98.88%, showing a relatively balanced performance. This suggests that the model has good prediction stability for samples of this category. Overall, the model exhibited good generalization ability and stability across different behavior categories.

**Figure 7:**
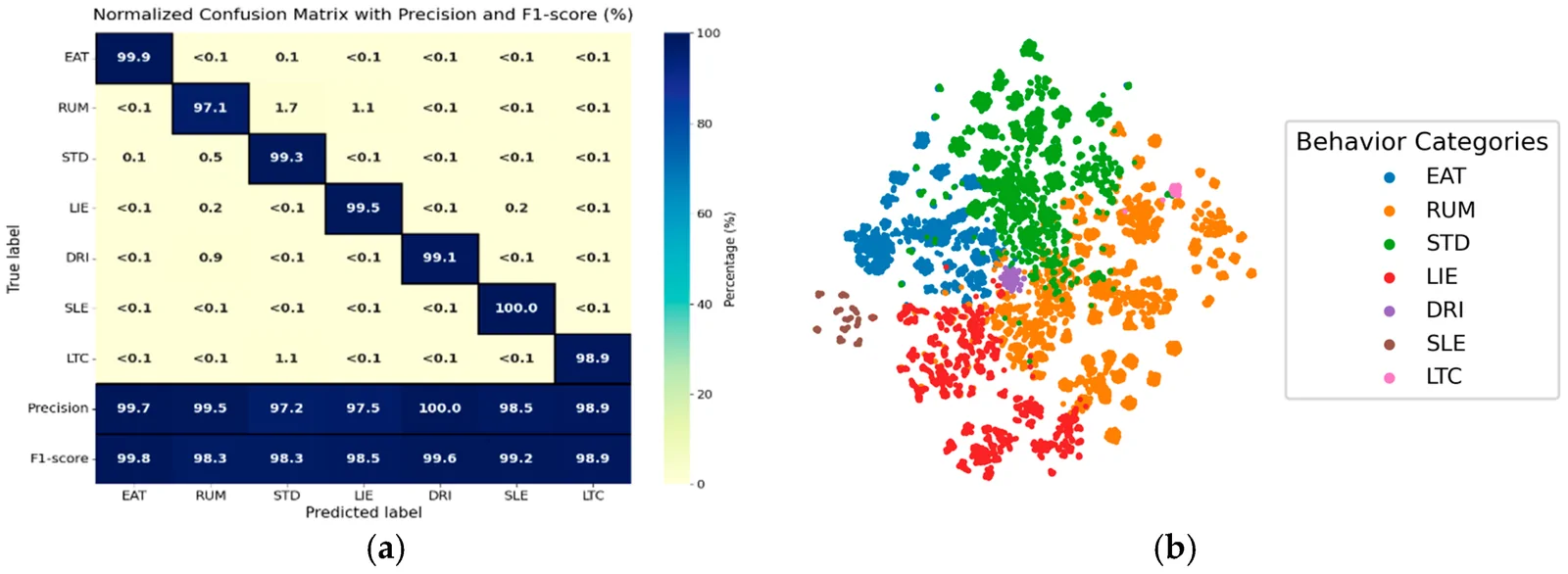
Confusion Matrix and Visualization of Fused Features. (**a**) Confusion matrix plot; (**b**) Feature visualization results.

To intuitively demonstrate the model’s discriminative performance across different dairy cow behavior categories and the potential confusion among them, a confusion matrix was plotted, as shown in Figure 7. Furthermore, to analyze the model’s classification capability from the perspective of feature representation, this study extracted the deep fusion features of the validation samples before the classifier and applied t-distributed stochastic neighbor embedding (t-SNE) to reduce them to a two-dimensional space for visualization. The results are shown in Figure 7.

As shown in the confusion matrix, most samples are concentrated along the diagonal, indicating that the model achieves high overall classification accuracy and that misclassification among different categories is relatively limited. In particular, the prediction results for behaviors such as eating, drinking, and sleeping are the most concentrated, with almost no obvious confusion observed. This indicates that the dual-branch model can effectively address the problem that similar behaviors are easily confused when only single-source IMU information is used.

As shown in the fused feature visualization, different behavior categories exhibit clear clustering and separation trends in the two-dimensional feature space, indicating that the deep fused features learned by the model have strong category discrimination ability. Among them, lying, standing, ruminating, and eating form relatively independent cluster regions, while minority-sample categories such as drinking, sleeping, and lateral trunk contact also show distinguishable local clusters. This suggests that the model has good feature extraction capability and recognition ability for minority-sample categories. These results further verify the effectiveness and stability of the model from the perspective of feature space.

### 3.2. Comparative Experiments of Different Models

To verify the effectiveness of the proposed model, it was compared with five representative models: a convolutional neural network (CNN), a long short-term memory network (LSTM), a hybrid CNN–LSTM model, a Transformer model, and a support vector machine (SVM). To ensure experimental fairness, all models were trained and tested using the same dataset and the same 10-fold cross-validation strategy. For the deep learning models, the number of training epochs was set to 50, the batch size was set to 64, and the Adam optimizer with the OneCycle learning-rate scheduling strategy was adopted.

**Figure 8:**
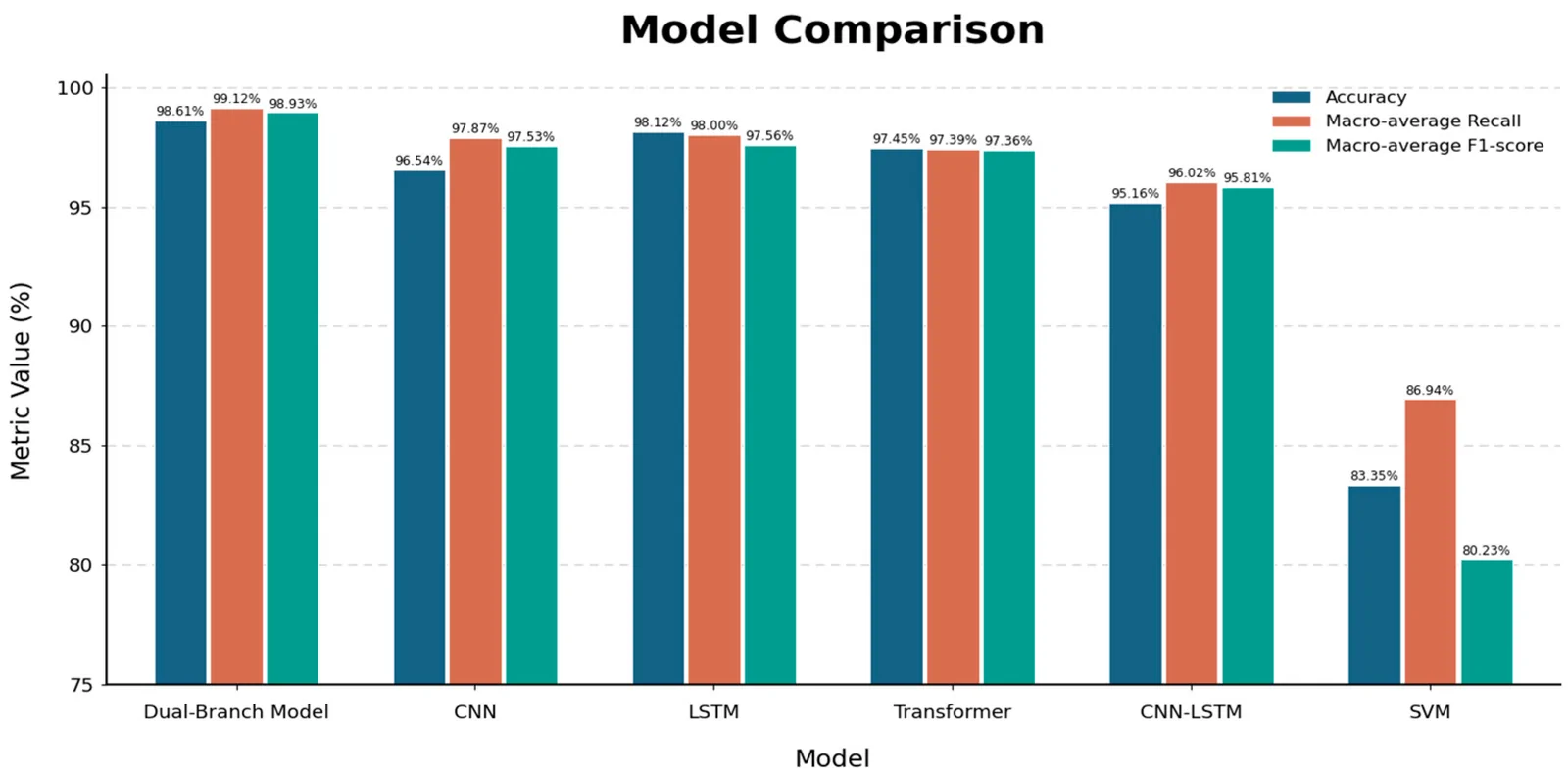
Comparison of Model Parameters.

Accuracy, macro-average recall, and macro-average F1-score were used as the evaluation metrics for all models. The overall performance was evaluated based on the prediction results obtained on the validation sets, and the results are shown in Figure 8. As shown in the figure, all six models achieved satisfactory recognition performance, with the deep learning models generally outperforming the SVM model. The proposed dual-branch gated fusion model achieved the best performance across all three metrics, with an accuracy, macro-average recall, and macro-average F1-score of 98.61%, 99.12%, and 98.93%, respectively. Compared with the CNN model, these three metrics increased by 2.07, 1.25, and 1.40 percentage points, respectively; compared with the LSTM model, they increased by 0.49, 1.12, and 1.37 percentage points, respectively. Furthermore, compared with the CNN–LSTM model, the proposed model improved the three metrics by 3.45, 3.10, and 3.12 percentage points, respectively, while compared with the Transformer model, the corresponding improvements were 1.16, 1.73, and 1.57 percentage points.

The results show that the proposed model further improves the macro-average recall and F1-score while maintaining high recognition accuracy. This indicates that the model not only has strong overall classification capability, but also achieves more balanced recognition performance across different categories, thereby verifying its effectiveness and superiority.

### 3.3. Ablation Study

#### 3.3.1. Analysis of Evaluation Metrics

To further verify the effectiveness of each key module in the constructed dual-branch model, four groups of ablation experiments were designed to investigate the effects of introducing the UWB branch (M1) and the SE attention mechanism module (M2) on the recognition performance of the model. The results are shown in Table 4. In the table, “√” indicates that the corresponding module is used, while “×” indicates that the module is not used.

As shown in Table 4, when the baseline model A does not incorporate the M1 and M2 modules, its accuracy, macro-average recall, and macro-average F1-score are 94.76%, 96.51%, and 93.07%, respectively. After introducing only the UWB branch, these metrics increase to 97.56%, 98.64%, and 98.19%, representing improvements of 2.80, 2.13, and 5.12 percentage points over model A, respectively. This indicates that the UWB branch can effectively alleviate the limitations caused by insufficient feature representation in the original model and enhance the model’s ability to distinguish samples from different categories.

For model C, which introduces only the SE attention mechanism, the accuracy, macro-average recall, and macro-average F1-score reach 95.48%, 96.57%, and 94.07%, respectively. Although these results show certain improvements over model A, the gains are relatively limited. This suggests that the SE module can improve recognition performance by adaptively weighting channel features, strengthening key discriminative information, and suppressing redundant features; however, its individual effect is weaker than that of the UWB branch.

Furthermore, model D, which simultaneously incorporates M1 and M2, achieves the best performance, with accuracy, macro-average recall, and macro-average F1-score reaching 98.61%, 99.12%, and 98.93%, respectively. Compared with model B, these values further increase by 1.05, 0.48, and 0.74 percentage points, respectively; compared with model C, they increase by 3.13, 2.55, and 4.86 percentage points, respectively. The results demonstrate that the UWB branch is the primary factor contributing to model performance improvement, while the SE attention mechanism further enhances feature selection and representation capabilities. Their synergistic effect enables the model to achieve the best recognition performance, verifying the rationality and effectiveness of the dual-branch fusion model architecture.

#### 3.3.2. Confusion Matrix Analysis

**Figure 9:**
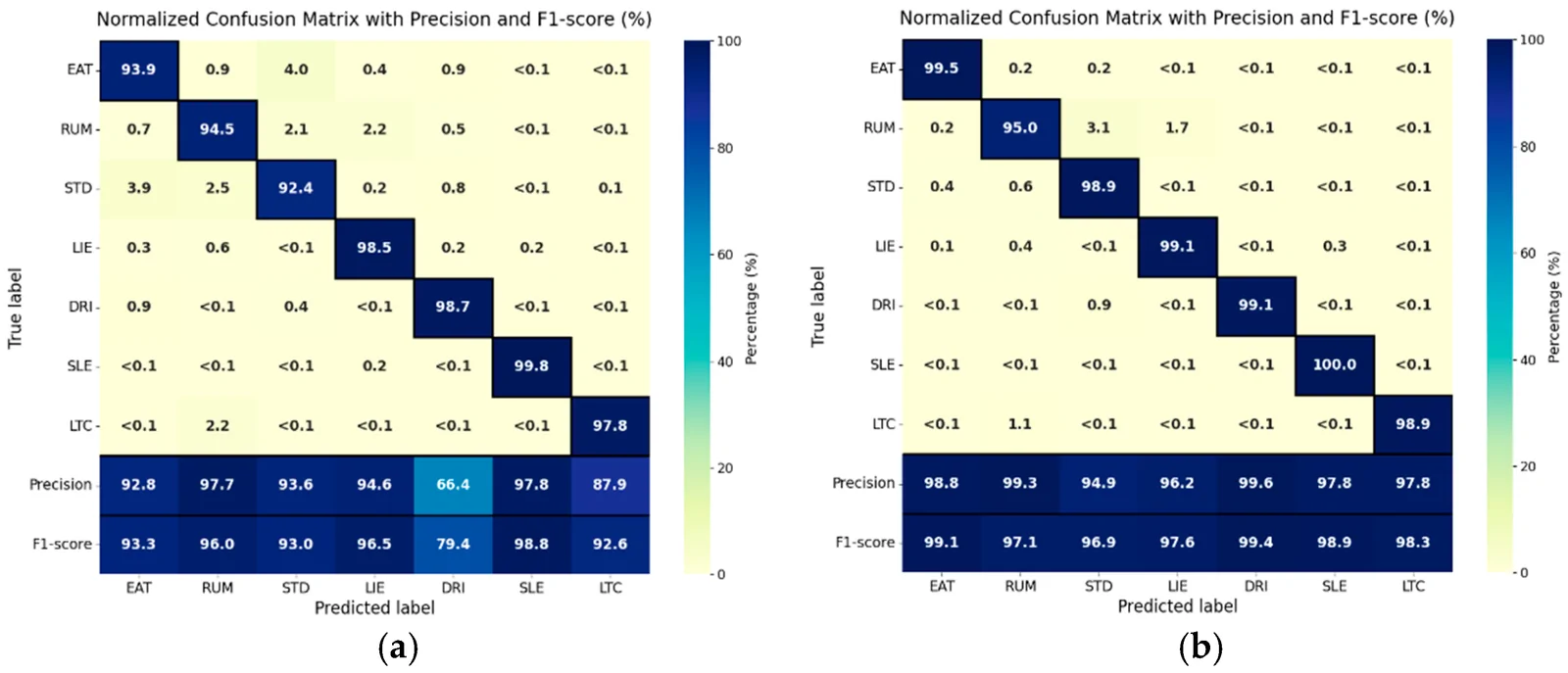

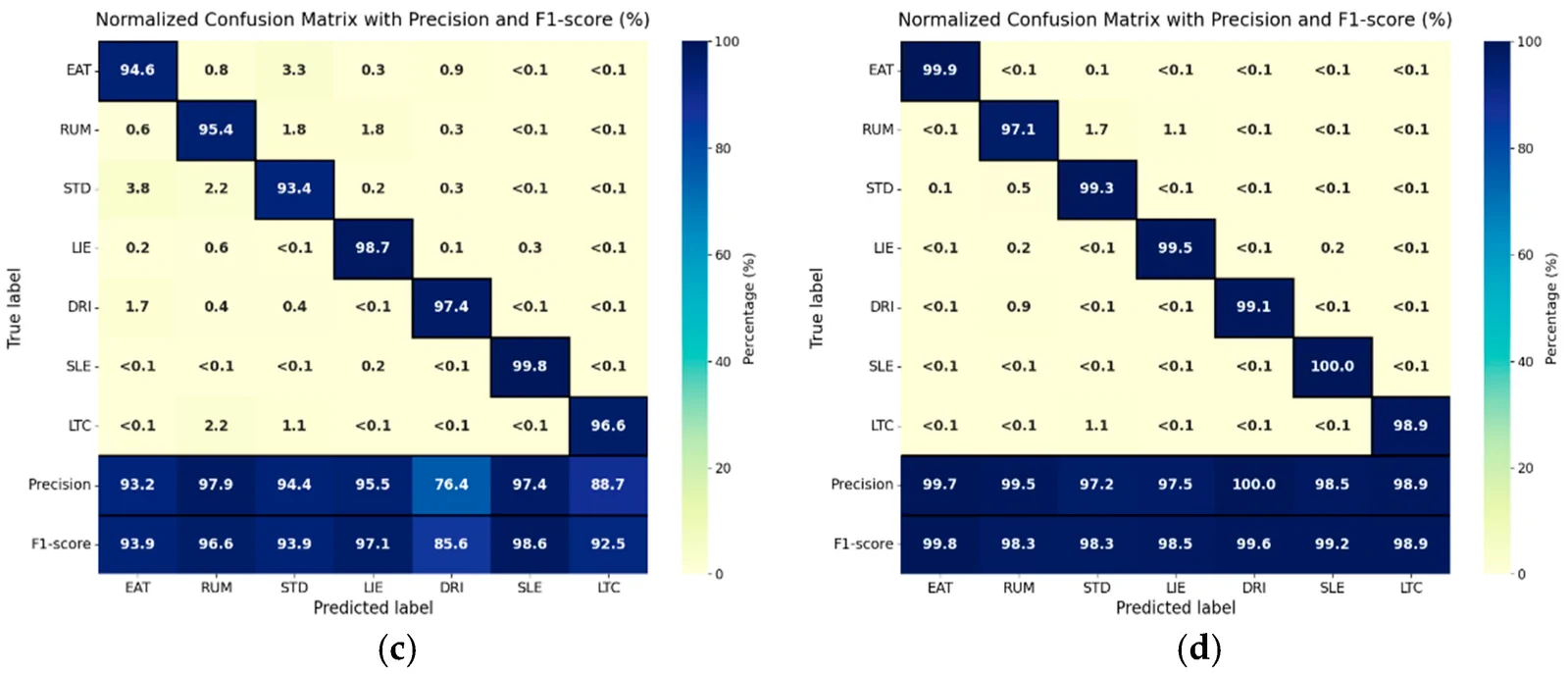
Comparison of Confusion matrices. (**a**) Model A; (**b**) Model B; (**c**) Model C; (**d**) Model D.

Although accuracy, recall, and F1-score can reflect model performance from an overall perspective, they are still insufficient to reveal differences in the discriminative ability of different models across individual categories. To further analyze the influence of each module on inter-class confusion, this study plots confusion matrices under different settings. The confusion matrices are generated based on the out-of-fold (OOF) prediction results obtained from 10-fold cross-validation. Each sample is predicted without participating in the corresponding training process, as shown in the following Figure 9.

As shown in the figure above, the baseline model A exhibits relatively obvious confusion in several categories. Specifically, standing (STD) is misclassified as eating (EAT) and ruminating (RUM) at rates of 3.9% and 2.5%, respectively, while lateral trunk contact (LTC) is misclassified as ruminating at a rate of 2.2%. The precision of the drinking (DRI) category is only 66.4%, indicating that relying solely on IMU information is insufficient for distinguishing similar behaviors and behaviors with limited samples.

After introducing only the SE attention mechanism, model C shows certain improvements. However, standing is still misclassified as eating and ruminating at rates of 3.8% and 2.2%, respectively, and the precision of drinking increases only to 76.4%. This indicates that the SE module mainly serves to enhance feature representation, while its ability to independently reduce inter-class confusion is limited.

After introducing the UWB branch, inter-class confusion in model B is significantly alleviated. The precision of drinking increases substantially from 66.4% to 99.6%, demonstrating that UWB spatial position information can effectively compensate for the limitations of IMU temporal features in distinguishing similar behaviors.

Furthermore, model D achieves the best performance. The misclassification rates of standing as eating and ruminating are further reduced to 0.1% and 0.5%, respectively, while the recognition rate of sleeping (SLE) reaches 100.0%. These results show that the proposed dual-branch fusion model, which combines UWB spatial information supplementation with SE attention enhancement, can effectively reduce confusion among similar behaviors and significantly improve the recognition performance of minority-sample categories, thereby verifying the effectiveness of the model architecture.

## 4. Discussion

### 4.1. Performance and Innovation Analysis of the Proposed Method

The proposed IMU–UWB dual-branch fusion model achieved an accuracy of 98.61%, a macro-averaged recall of 99.12%, and a macro-averaged F1-score of 98.93%, demonstrating strong and well-balanced classification performance in dairy cow behavior recognition. Under the same validation strategy, the proposed model outperformed the CNN, LSTM, CNN–LSTM, Transformer, and SVM models across all three evaluation metrics: accuracy, macro-averaged recall, and macro-averaged F1-score. In particular, the improvements in macro-averaged recall and macro-averaged F1-score indicate that the proposed method not only improves overall recognition accuracy but also maintains relatively stable performance across behavioral categories with substantially different sample sizes.

The ablation experiments further clarified the specific contributions of the UWB branch and the SE attention mechanism to model performance. After incorporating the UWB branch into the baseline model, the macro-averaged F1-score increased from 93.07% to 98.19%, indicating that the spatial distance information provided by UWB was the primary contributor to the performance improvement. In comparison, introducing only the SE mechanism resulted in a relatively limited improvement. This is because the SE mechanism mainly enhances key motion features and suppresses redundant information by adaptively weighting different IMU channels, but does not introduce an additional source of behavioral information. When the UWB branch and the SE mechanism were incorporated simultaneously, the macro-averaged F1-score further increased to 98.93%. This result demonstrates the strong complementarity between UWB spatial information and IMU channel-level feature enhancement: the former provides spatial and positional cues associated with behavioral activities, whereas the latter optimizes the representation of existing motion features, thereby jointly improving the discriminative capability of the model.

The confusion matrix results further confirmed this complementarity. In the baseline model, standing behavior was misclassified as eating and rumination at rates of 3.9% and 2.5%, respectively, whereas these rates decreased to 0.1% and 0.5% in the complete model. In addition, after the introduction of UWB information, the precision for drinking recognition increased markedly from 66.4% to 99.6%. These findings indicate that relying solely on neck-mounted IMU motion features makes it difficult to distinguish behaviors with similar postures or movement patterns, whereas UWB spatial distance information provides additional environmental and positional cues, effectively reducing confusion among similar behaviors. On this basis, the SE mechanism further strengthens discriminative IMU channel features, enabling the model to maintain stable recognition performance for minority-sample behaviors such as drinking, sleeping, and lateral trunk contact.

While achieving high recognition performance, the proposed model retains a lightweight architecture, with 0.384 M parameters, 0.013 GFLOPs, and a model storage size of 1.469 MB. Compared with multimodal approaches that combine inertial sensing information with images, sound, or multiple physiological signals [25,26,27], the proposed framework relies only on neck-mounted inertial signals and ultra-wideband ranging data and employs two lightweight feature-extraction branches. This design reduces the model’s processing complexity and is therefore more suitable for deployment on resource-constrained edge devices. Liu et al. [28] previously demonstrated the feasibility of combining IMU and UWB data for behavior classification and movement analysis. Building on their work, the present study explicitly models the spatial-distance features provided by UWB data through a dedicated branch and adaptively integrates them with IMU motion features using a gating mechanism. The ablation results showed that introducing the UWB branch led to clear improvements in both classification accuracy and the macro-averaged F1-score, indicating that the spatial-distance information was effectively utilized. Therefore, the proposed model not only demonstrates strong behavior-recognition capability but also has the potential for deployment on edge devices and continuous real-time monitoring. It can provide reliable behavioral information to support dairy cow health assessment, abnormal behavior warning, welfare evaluation, and precision feeding management.

### 4.2. Limitations and Future Work

Although the proposed method achieves high recognition accuracy, some limitations remain. First, the experimental data were collected from only four healthy dairy cows on the same farm over a six-day period, and the diversity of experimental subjects and scenarios remains limited. Although a relatively large number of window samples were generated, these samples cannot fully compensate for the limited number of biologically independent animals and the short monitoring period. In addition, stratified 10-fold cross-validation was performed randomly at the window-sample level rather than at the animal level. Consequently, samples from the same cow may have appeared in both the training and validation sets, which could have resulted in overly optimistic performance estimates. The generalization ability of the model across different farms, breeds, physiological stages, and feeding environments requires further validation. Second, the sample distribution among different behavior categories is imbalanced, with relatively few samples for drinking, sleeping, and lateral trunk contact. Although a class-weighted cross-entropy loss was adopted during model training to mitigate the influence of class imbalance, this strategy does not increase the actual number or behavioral diversity of minority-class samples. Therefore, the stable recognition and generalization of low-frequency behaviors in practical applications may still be affected. Future research will further expand the dataset, increase the number of experimental animals, extend the monitoring period, and explore real-time deployment of the model on edge devices to improve the applicability and robustness of the proposed method in actual dairy farming environments.

## 5. Conclusions

To address confusion among similar dairy cow behaviors when using IMU data alone, this study proposed an IMU–UWB dual-branch fusion model. The IMU branch extracts temporal motion features, while the UWB branch provides complementary spatial distance information. An SE attention mechanism was further introduced to enhance discriminative channel features and suppress redundant information. The model recognized seven dairy cow behaviors and achieved an accuracy of 98.61%, a macro-average recall of 99.12%, and a macro-average F1-score of 98.93%. Comparative and ablation experiments showed that UWB information reduced confusion among similar behaviors and improved the recognition of minority-sample categories, while the SE mechanism enhanced feature representation. Overall, the proposed method provides an effective approach for fine-grained dairy cow behavior monitoring and lays a methodological foundation for health assessment, abnormality warning, and precision farm management.

## Figures and Tables

**Table 1:** Sample information.

Class	Sample Size	Percentage
Eating (EAT)	3436	17.10 (%)
Ruminating (RUM)	7712	38.40 (%)
Standing (STD)	4729	23.55 (%)
Lying (LIE)	3434	17.10 (%)
Drinking (DRI)	232	1.16 (%)
Sleeping (SLE)	450	2.24 (%)
Lateral Trunk Contact (LTC)	89	0.44 (%)

**Table 2:** Model parameter performance.

Parameters	Results (%)
Average Precision	98.61%
Macro-Average Recall	99.12%
Macro-Average F1 Score	98.93%
Total Number of Parameters	0.384 M
Floating-Point Operations	0.013 GFLOPs
Model Memory Usage	1.469 MB

**Table 3:** Recognition results of different cow behaviors.

Class	Precision (%)	Recall (%)	F1 Score (%)
Eating	99.65	99.85	99.75
Ruminating	99.51	97.12	98.30
Standing	97.23	99.34	98.27
Lying	97.49	99.48	98.47
Drinking	100.00	99.14	99.57
Sleeping	98.47	100.00	99.23
Lateral Trunk Contact	98.88	98.88	98.88

**Table 4:** Results of ablation experiment.

Model	M1	M2	Average Precision (%)	Macro-Average Recall (%)	Macro-Average F1 Score (%)
A	×	×	94.76	96.51	93.07
B	√	×	97.56	98.64	98.19
C	×	√	95.48	96.57	94.07
D	√	√	98.61	99.12	98.93

## Data Availability

As the original data were collected from a commercial dairy farm, the data used in this study are available from the corresponding author upon reasonable request.

## Abbreviations

The following abbreviations are used in this manuscript:

CNN	Convolutional neural network
FCN	Fully convolutional network
FCN-SE	Fully convolutional network with squeeze-and-excitation modules
FLOPs	Floating-point operations
IMU	Inertial measurement unit
LSTM	Long short-term memory
SE	Squeeze-and-excitation
SVM	Support vector machine
t-SNE	t-distributed stochastic neighbor embedding
UWB	Ultra-wideband
